# Treatment Discontinuation in Patients With Muscle-Invasive Bladder Cancer Undergoing Chemoradiation

**DOI:** 10.1016/j.adro.2021.100836

**Published:** 2021-10-25

**Authors:** Nikhil V. Kotha, Abhishek Kumar, Tyler J. Nelson, Edmund M. Qiao, Alex S. Qian, Rohith S. Voora, Rana R. McKay, Tyler F. Stewart, Brent S. Rose

**Affiliations:** a Department of Radiation Medicine and Applied Sciences, University of California San Diego, La Jolla, California; b Veterans Affairs San Diego Healthcare System, San Diego, California; c Department of Radiation Oncology, Duke University, Durham, North Carolina; d Division of Hematology-Oncology, Department of Medicine, University of California San Diego, La Jolla, California

## Abstract

**Purpose:**

Chemoradiation (CRT) is a definitive treatment option for muscle-invasive bladder cancer (MIBC). Despite its effectiveness, CRT is underused, in part owing to concerns of tolerability and the need for integrated multidisciplinary care. We investigated factors associated with and the impact of treatment discontinuation in patients with MIBC treated with CRT.

**Methods and Materials:**

In the US Veterans Affairs’ national database, we identified patients with urothelial histology, MIBC (T2-4a/N0-3/M0) diagnosed between 2000 and 2018 and treated with definitive-intent CRT. The primary endpoint of discontinued radiation was evaluated in a multivariable logistic regression. Secondary endpoints of 30-day and 90-day mortality, overall mortality, and nonbladder cancer mortality were evaluated in multivariable models.

**Results:**

Of 369 veterans with MIBC who underwent CRT, 30 patients (8.1%) did not complete radiation. The most common reasons for treatment discontinuation included comorbidities or infections necessitating hospital admission (63.3%) and treatment intolerance or declining performance status (26.7%). In multivariable logistic regression, variables associated with radiation discontinuation were creatinine clearance ≤ 50 (odds ratio [OR], 3.93; 95% CI, 1.63-9.50; *P* = .002), incomplete transurethral resection of bladder tumor (TURBT) (OR, 3.16; 95% CI, 1.15-8.63; *P* = .02), and nonpreferred chemotherapy (OR, 3.31; 95% CI, 1.31-8.36; *P* = .01). In the cohort that discontinued radiation, 30-day mortality was 33.3% and 90-day mortality was 50.0%, with the majority of deaths attributed to nonbladder cancer causes. No patient or tumor variables were associated with either endpoint. In the cohort that completed radiation, 30-day mortality was 2.7% and 90-day mortality was 6.8%. In multivariable analysis, radiation discontinuation was associated with worse overall mortality (hazard ratio [HR], 2.48; 95% CI, 1.36-4.50; *P* = .003) and worse nonbladder cancer mortality (HR, 2.32; 95% CI, 1.24-4.34; *P* = .008).

**Conclusions:**

With a low rate of treatment discontinuation, CRT is an effective and feasible treatment option for the typically elderly and comorbid population of patients with MIBC. In addition to identified predictors of treatment discontinuation (poor renal function, incomplete TURBT, etc.), further research is required to develop evidence-based guidelines for optimal patient selection.

## Introduction

Muscle-invasive bladder cancer (MIBC) is an aggressive disease that predominantly affects elderly patients (60-80 years old) and requires definitive treatment to prevent progression and cancer-related symptoms and death. Radical cystectomy with or without neoadjuvant chemotherapy and radiation therapy with concurrent radiosensitizing chemotherapy (CRT) are definitive treatment options for MIBC that have been used to varying degrees.^1^^,^^2^ Concurrent radiosensitizing chemotherapy has a favorable toxicity profile, especially in relation to the morbidity and life-altering bladder removal or replacement in radical cystectomy, and may be an ideal treatment strategy for an elderly population with comorbidities.^3^^,^^4^ Despite these options, 33% to 50% of all patients with MIBC do not undergo any definitive treatment,^2^^,^^5^^,^^6^ and only 10% of patients with MIBC are treated with CRT.^7^^,^^8^

Given that MIBC typically occurs in older individuals who frequently have concurrent medical comorbidities, we hypothesized that CRT is not pursued more often in part owing to concerns for patient tolerance with treatment.^9^ Concurrent radiosensitizing chemotherapy requires a minimum duration of 4 weeks of daily radiation treatment in conjunction with chemotherapy, which carries its own inherent risks for toxic effects and tolerability. Furthermore, literature on CRT for MIBC is limited outside of single-arm studies composed of ideally selected, healthier patients compared with the respective real-world population. We investigated radiation treatment completion rates, the factors associated with discontinuation, and the impact of treatment discontinuation in patients with MIBC treated with CRT within the US Veterans Affairs’ (VA) database.

## Methods

### Data source

Veterans Affairs Informatics and Computing Infrastructure (VINCI) is a comprehensive informatics platform that enables access to the VA's national database composed of patient-level electronic health records and administrative data. VINCI includes access to more than 21 million veterans’ inpatient and outpatient data across 152 nationwide medical centers.^10^ Tumor registry data are uploaded by trained registrars in accordance with protocols issued from the American College of Surgeons, thereby capturing an estimated 90% of incident cancers within the VA system.^11^^,^^12^ Cause-specific mortality information (*International Statistical Classification of Diseases and Related Health Problems, Tenth Revision (ICD-10)* code C67 for bladder cancer) was obtained from the National Death Index. Our protocol was approved by the San Diego VA Institutional Review Board. Informed signed consent was waived by the institutional review board given that this was a retrospective analysis with minimal risk to the rights and welfare of participants and one that could not practically be completed without the waiver.

### Patient selection and covariables

From VINCI, we identified 407 veterans with histologically confirmed, localized muscle-invasive urothelial carcinoma (T2-4a, N0-3, M0) diagnosed between 2000 and 2018 who were treated with definitive-intent CRT. Successful definitive CRT was defined as a radiation dose of at least 55 Gy in conjunction with at least 1 cycle of chemotherapy administered within 14 days of the radiation start date. For patients who did not finish definitive radiation, the reason for discontinuation was recorded. Patients were excluded for absent follow-up records or unknown cause of death. The final cohort consisted of 369 patients.

Covariables of interest included sociodemographic variables, Charlson Comorbidity Index, creatinine clearance (estimated by the Cockcroft-Gault equation)^13^ before CRT, T/N categories according to the American Joint Committee on Cancer eighth edition staging system, presence of pretreatment hydronephrosis, visibly complete or successful resection of tumor after transurethral resection of bladder tumor (TURBT) according to operative reports before CRT, and type of concurrent chemotherapy agent. The National Comprehensive Cancer Network's recommendations for “preferred” regimens (cisplatin alone, cisplatin and paclitaxel, cisplatin and fluorouracil, and mitomycin and fluorouracil) were used for categorization of chemotherapy.^14^ All variables and outcomes were manually chart reviewed if not present within database elements. All patients were followed until death or the last follow-up with a VA practitioner before January 1, 2021.

### Outcomes and statistical analysis

The primary endpoint was the discontinuation rate among patients receiving definitive-intent CRT. Secondary endpoints included 30-day and 90-day (posttreatment) mortality, overall mortality (OM), and nonbladder cancer mortality (NCM), defined using a competing risk analysis framework with bladder cancer–specific mortality (BCM) as a competing risk. We calculated OM and NCM from the date of diagnosis. Discontinued radiation and 30-day and 90-day mortality were evaluated in multivariable logistic regressions. Baseline characteristics were compared between the cohorts that completed treatment and discontinued treatment using the χ^2^ test and Wilcoxon rank sum test as appropriate. Overall survival was assessed with Kaplan-Meier analysis, OM was assessed with Cox proportional hazards regression analysis, and NCM was assessed with cumulative incidence analysis and Fine-Gray regression analysis. All multivariable models were chosen a priori*.* All variables in multivariable models were confirmed to have no collinearity or interactions. Odds ratios (ORs) for logistic analysis or hazard ratios (HRs) for survival analysis and respective 95% confidence intervals (CIs) are reported. All statistical analyses were performed using SAS, version 9.4 (SAS Institute, Cary, North Carolina), with 2-sided *P* values less than .05 considered statistically significant.

## Results

### Baseline characteristics

Of 369 patients with MIBC who were planned to receive definitive-intent CRT, 30 patients (8.1%) did not complete definitive radiation. In the discontinued treatment cohort, the median radiation dose received was 36.0 Gy (range, 5.4-50.4 Gy).

The most common reason for treatment discontinuation was comorbidities or infections necessitating hospital admission (19 patients [63.3%]) — 3 cases of urinary tract infection, 3 cases of pneumonia, 3 cases of sepsis of unknown origin, 2 cases of *Clostridium difficile* infection, 2 cases of acute renal failure, 3 cases of cardiopulmonary problems (heart failure, respiratory failure, and myocardial infarction), and 3 cases of exacerbations of miscellaneous comorbidities. The next most common reasons for treatment discontinuation were treatment intolerance or declining performance status (8 patients [26.7%]), patient decision (1 patient [3.3%]), proximity of small bowel (1 patient [3.3%]), and discovery of metastases during treatment (1 patient [3.3%]).

For the overall cohort, the median age was 78 years, with 70.7% of patients older than 70 years and 34.2% of patients older than 80 years. Compared with the cohort that completed treatment, patients in the cohort that discontinued treatment were less likely to be active smokers at diagnosis (16.7% vs 35.1%), more likely to have creatinine clearance ≤50 (66.7% vs 36.3%), more likely to have visibly incomplete TURBT (26.7% vs 12.4%), and more likely to receive nonpreferred chemotherapy (76.7% vs 46.0%) (Table 1). The rest of the baseline characteristics, including age and comorbidities, were broadly similar between the 2 cohorts. Of note, the overall cohort's active smokers at diagnosis were younger (median age, 69 vs 78 years; *P* < .001) and had similar Charlson Comorbidity Index scores (*P* = .73) compared with nonactive smokers at diagnosis.

**Table 1:** Baseline patient, tumor, and treatment variables of each treatment cohort

Variable	Discontinued treatment, no. (%) (n = 30)	Completed treatment, no. (%) (n = 339)	*P* value
Age, median (range)	78 (62-90)	76 (53-93)	.35
Gender			.60
Male	30 (100)	336 (99.1)	
Female	0 (0)	3 (0.9)	
Race			.83
White	27 (90.0)	309 (91.2)	
Black, Asian, or Other	3 (10.0)	30 (8.8)	
Charlson Comorbidity Index score			.36
0	10 (33.3)	87 (25.7)	
1+	20 (66.7)	252 (74.3)	
Smoker at diagnosis			.04
Yes	5 (16.7)	119 (35.1)	
No	25 (83.3)	220 (64.9)	
Married			.34
Yes	14 (46.7)	189 (55.8)	
No	16 (53.3)	150 (44.2)	
Median income in zip code, US dollars, thousands			.50
<50	15 (50.0)	191 (56.3)	
≥50	15 (50.0)	148 (43.7)	
Percentage of population with a bachelor's degree in zip code			.92
≤15	16 (53.3)	184 (54.3)	
>15	14 (46.7)	155 (45.7)	
Creatinine clearance			.001
≤50	20 (66.7)	123 (36.3)	
>50	10 (33.3)	216 (63.7)	
T Category			.53
2	24 (80.0)	286 (84.4)	
3-4	6 (20.0)	53 (15.6)	
N Category			.73
0	29 (96.7)	323 (95.3)	
1-3	1 (3.3)	16 (4.7)	
Pretreatment hydronephrosis			
Yes	13 (43.3)	112 (33.0)	.25
No	17 (56.7)	227 (67.0)	
Successful TURBT			.03
Yes	22 (73.3)	297 (87.6)	
No	8 (26.7)	42 (12.4)	
Chemotherapy regimen			.001
Preferred	7 (23.3)	183 (54.0)	
Nonpreferred	23 (76.7)	156 (46.0)	

Abbreviation: TURBT = transurethral resection of bladder tumor.

### Radiation discontinuation analysis

In multivariable logistic regression, the variables associated with increased odds of radiation discontinuation were creatinine clearance ≤50 (OR, 3.93; 95% CI, 1.63-9.50; *P* = .002), visibly incomplete TURBT (OR, 3.16; 95% CI, 1.15-8.63; *P* = .02), and nonpreferred chemotherapy (OR, 3.31; 95% CI, 1.31-8.36; *P* = .01) (Table 2). Being an active smoker at diagnosis was associated with decreased odds of radiation discontinuation (OR, 0.30; 95% CI, 0.10-0.91; *P* = .03). Of note, older age, presence of comorbidities, residence in less affluent or less educated zip codes, presence of advanced T or N category disease, and presence of pretreatment hydronephrosis were not associated with radiation discontinuation. In sensitivity analyses replicating the illustrated multivariable model except with age classified as categorical (cutoff points of 70, 75, and 80 years), age continued to not be associated with radiation discontinuation. In sensitivity analyses replicating the illustrated multivariable model except with the Charlson Comorbidity Index score classified as 0, 1, or 2+ or as a continuous variable, the Charlson Comorbidity Index score continued to not be associated with radiation discontinuation.

**Table 2:** Logistic regression on radiation treatment completion

Variable	Radiation treatment completion	
	OR (95% CI)	*P* value
Age	0.96 (0.90-1.02)	.16
Sex Male Female	1.00 <0.01 (0.01-99.99)	.99
Race White Black, Asian, or Other	1.00 0.93 (0.24-3.65)	.92
Charlson Comorbidity Index score 0 1+	1.00 0.68 (0.29-1.61)	.38
Smoker at diagnosis No Yes	1.00 0.30 (0.10-0.91)	.03
Married No Yes	1.00 0.54 (0.24-1.22)	.14
Median income in zip code, US dollars, thousands <50 ≥50	1.00 1.81 (0.69-4.75)	.23
Percentage of population with a bachelor's degree in zip code ≤15 >15	1.00 0.72 (0.28-1.86)	.49
Creatinine clearance >50 ≤50	1.00 3.93 (1.63-9.50)	.002
T Category 2 3-4	1.00 1.20 (0.43-3.35)	.73
N Category 0 1-3	1.00 0.60 (0.07-5.18)	.64
Pretreatment hydronephrosis No Yes	1.00 0.91 (0.39-2.13)	.82
Successful TURBT Yes No	1.00 3.16 (1.15-8.63)	.02
Chemotherapy regimen Preferred Nonpreferred	1.00 3.31 (1.31-8.36)	.01

Abbreviations: CI = confidence interval; OR = odds ratio; TURBT = transurethral resection of bladder tumor.

### Survival analysis

The median follow-up time was 77 months. In the cohort that discontinued radiation, 30-day mortality was 33.3% (10 patients) and 90-day mortality was 50.0% (15 patients), with the majority of deaths attributed to nonbladder cancer causes. In multivariable models, no patient, tumor, or treatment variables were associated with either 30-day or 90-day mortality (data not shown). In the cohort that completed radiation, these endpoints were numerically better; 30-day mortality was 2.7% and 90-day mortality was 6.8%.

**Fig. 1:**
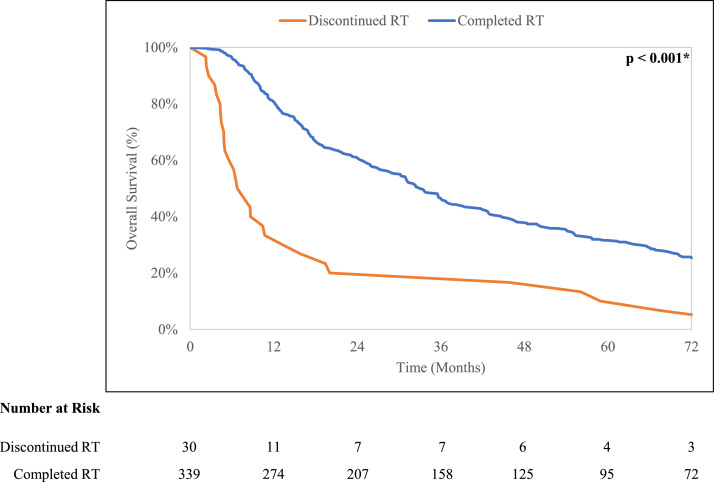
Kaplan-Meier curves for overall survival stratified by treatment completion.

**Fig. 2:**
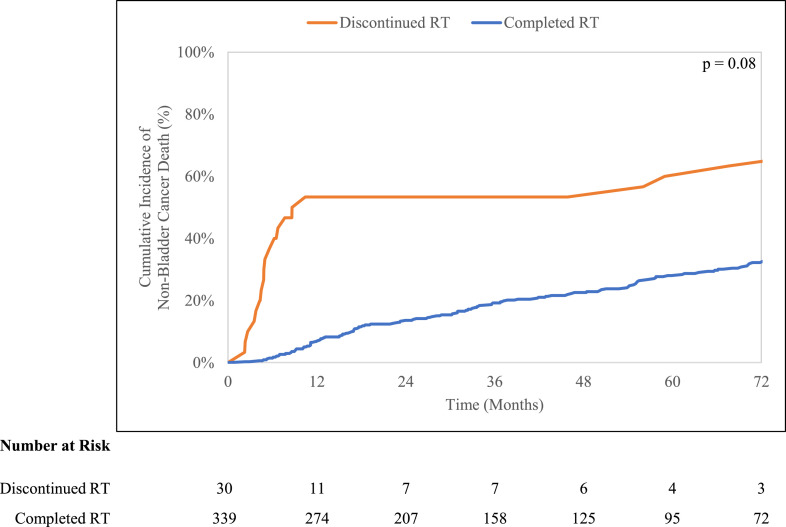
Cumulative incidence curves for nonbladder cancer mortality stratified by treatment completion.

Improved overall survival (*P* < .001) was observed for the cohort that completed radiation (6-year overall survival, 25.7%; 95% CI, 21.0%-30.6%) compared with the cohort that discontinued radiation (6-year overall survival, 6.7%; 95% CI, 1.1%-19.1%) (Fig 1). There was a trend toward significantly worse NCM (*P* = .08) for the cohort that discontinued radiation (6-year NCM, 63.3%; 95% CI, 42.3%-78.5%) compared with the cohort that completed radiation (6-year NCM, 32.2%; 95% CI, 27.2%-37.4%) (Fig 2). No significant difference (*P* = .22) in BCM was observed (completed cohort: 6-year BCM, 42.1%; 95% CI, 36.8%-47.3%; discontinued cohort: BCM, 30.0%; 95% CI, 14.4%-47.3%). In multivariable analysis, radiation discontinuation was significantly associated with worse OM (HR, 2.48; 95% CI, 1.36-4.50; *P* = .003) and worse NCM (HR, 2.32; 95% CI, 1.24-4.34; *P* = .008) (Table 3). Radiation discontinuation was not associated with BCM (HR, 0.56; 95% CI, 0.25-1.25; *P* = .16).

**Table 3:** Multivariable a priori regressions on overall mortality (OM) and nonbladder cancer mortality (NCM) in the overall cohort

	OM		NCM	
Variable	HR (95% CI)	*P* value	HR (95% CI)	*P* value
Radiation treatment Completed Discontinued	1.00 2.48 (1.36-4.50)	.003	1.00 2.32 (1.24-4.34)	.008
Age	1.00 (0.98-1.01)	.73	1.01 (0.99-1.04)	.24
Gender Male Female	1.00 0.90 (0.13-6.00)	.91	1.00 0.00 (0.00-0.00)	<.001
Race White Black, Asian, or Other	1.00 0.85 (0.54-1.34)	.49	1.00 0.64 (0.33-1.24)	.19
Charlson Comorbidity Index score 0 1+	1.00 1.15 (0.88-1.51)	.31	1.00 0.99 (0.69-1.41)	.95
Smoker at diagnosis No Yes	1.00 0.95 (0.71-1.27)	.71	1.00 1.27 (0.88-1.83)	.21
Married No Yes	1.00 1.18 (0.94-1.49)	.17	1.00 1.11 (0.80-1.54)	.54
Median income in zip code, US dollars, thousands <50 ≥50	1.00 1.26 (0.98-1.62)	.07	1.00 1.13 (0.76-1.68)	.56
Percentage of population with a bachelor's degree in zip code ≤15 >15	1.00 0.84 (0.65-1.08)	.17	1.00 0.95 (0.63-1.41)	.78
Creatinine clearance >50 ≤50	1.00 1.35 (1.07-1.71)	.01	1.00 0.98 (0.69-1.41)	.93
T Category 2 3-4	1.00 1.28 (0.93-1.75)	.13	1.00 0.92 (0.58-1.48)	.74
N Category 0 1-3	1.00 3.20 (1.99-5.14)	<.001	1.00 0.75 (0.29-1.94)	.56
Pretreatment hydronephrosis No Yes	1.00 1.45 (1.14-1.85)	.003	1.00 0.81 (0.56-1.18)	.27
Successful TURBT Yes No	1.00 1.07 (0.77-1.50)	.69	1.00 1.25 (0.78-2.02)	.35
Chemotherapy regimen Preferred Nonpreferred	1.00 1.21 (0.96-1.52)	.10	1.00 1.62 (1.14-2.29)	.006

Abbreviations: CI = confidence interval; HR = hazard ratio; TURBT = transurethral resection of bladder tumor.

## Discussion

In this study of muscle-invasive bladder cancer treated with definitive-intent chemoradiation, the vast majority (> 90%) of patients were able to tolerate and complete a definitive radiation course. For the small percentage of patients who were not able to finish definitive radiation, factors associated with treatment discontinuation included decreased creatinine clearance, unsuccessful TURBT, and receipt of nonpreferred chemotherapy. Conversely, older age and comorbidities were not associated with radiation discontinuation. With a low rate of treatment discontinuation, the findings suggest that CRT is a feasible and promising definitive treatment option for the vast majority of the typically elderly and comorbid population of patients with MIBC.

The most common reasons for discontinuing radiation were comorbidities or infections and treatment intolerance or declining performance status. Given that these situations likely resulted from a combination of MIBC and increased competing mortality risk, the results of the multivariable analysis are contextualized. Multivariable models showed that rather than age or comorbidity score, the predictors of not completing radiation were those likely representative of invasive bladder cancer (resulting in decreased creatinine clearance, unsuccessful TURBT, and receipt of nonpreferred chemotherapy). However, it is possible that decreased creatinine clearance and receipt of nonpreferred chemotherapy were influenced by comorbidities in many of the patients. The significant association between being an active smoker at diagnosis and decreased odds of radiation discontinuation may be explained by the significant difference in age between the smoking cohorts. However, age was not associated with radiation discontinuation, and there was no significant interaction between age and smoking status in the multivariable models. Therefore, the observed paradoxical association between smoking and radiation discontinuation is likely explained by the combination of difference in age in conjunction with a potential “smoker's paradox”^15^, ^16^, ^17^ in this population but this requires further investigation. Alongside validating the identified variables in our analysis, additional markers should be sought to help accurately guide who will likely finish CRT and should therefore undergo definitive treatment.

In the cohort that discontinued treatment, the majority of the 30-day and 90-day mortality was attributed to nonbladder cancer causes. We hypothesize that owing to their competing mortality risk, either these patients had a minimal reserve of fitness left to tolerate treatment or they were close to dying before starting CRT. Although this study's models did not identify any sociodemographic or clinical markers predictive of death within 30 or 90 days after CRT, additional markers should be sought to help predict noncompeting mortality risk. Stratification tools may be beneficial to clinical practice by optimizing patient selection and ensuring treatment for those most likely to derive benefit. Additionally, although our survival analysis showed that radiation discontinuation was not associated with inferior BCM, this was likely owing to the majority of nonbladder cancer mortality in the discontinuation cohort and a consequent lack of bladder cancer–specific deaths. Further examination of local recurrences in this discontinued radiation cohort is warranted given significant literature in other cancer sites showing that radiation treatment interruption or discontinuation was associated with inferior recurrence and survival outcomes.^18^^,^^19^ Finally, given the significant association of radiation discontinuation with worse OM and NCM, the event of radiation discontinuation could be used as a signal to redefine goals of care and integrate additional multidisciplinary team members (palliative care, social work, etc.) to best provide end-of-life care.

Treatment decision making for patients with MIBC is multifactorial and can be difficult given the typically elderly and comorbid population in conjunction with the intensive nature and associated risk of complications with either radical cystectomy or CRT. Compared with radical cystectomy, CRT avoids the morbidity of surgery and the life-altering requirement of bladder removal and replacement.^3^^,^^4^ However, CRT is not without risk of toxic effects, because short-term bladder and bowel adverse effects are common.^3^^,^^20^ In addition, with a minimum duration of 4 weeks of daily treatments, it is easy to understand physician and/or patient hesitance to undergo CRT. To our knowledge, this study stands as one of the largest and most granular multi-institutional analyses to examine treatment discontinuation in patients with MIBC undergoing CRT. The results are generally consistent with those in previous literature from small, single-institution studies.^18^^,^^21^, ^22^, ^23^ In the landmark Bladder Cancer 2001 trial investigating the addition of chemotherapy to radiation therapy for MIBC, 95.1% of the CRT patients completed a definitive radiation regimen, slightly better than in this study.^24^ As expected in patients enrolled to clinical trials compared with those treated in routine clinical practice,^25^ the median age (72 years) of the trial's 182 patients was younger than the median age of this study's cohort. Also, given the trial's strict inclusion criteria (performance status 0-2, laboratory cutoff values, etc), the real-world population with MIBC captured in this study is likely not well represented in the trial's population. In spite of this, the overwhelming majority of patients in this study's cohort were able to complete radiation treatment. Furthermore, many of the patients in this study possessed features that are classically not ideal for bladder preservation treatment as per guidelines.^14^^,^^26^ These nonideal features include advanced T/N category disease, presence of hydronephrosis, and poor renal function. Despite the notable presence of many of these features in this study's cohort, more than 90% of patients were able to complete definitive radiation. Therefore, we posit that although there are “ideal” candidates for CRT, “nonideal” candidates as per current guidelines should still be considered for treatment. In light of the fact that 33% to 50% of patients with MIBC (and as high as 65% in patients aged 80 years or older) in the US and abroad do not undergo any definitive treatment,^2^^,^^5^^,^^6^^,^^27^^,^^28^ we hope this analysis of CRT outcomes in a typical, real-world population with MIBC provides useful information to patients and clinicians to discuss CRT as a viable treatment option for more individuals with MIBC (especially the elderly and those with comorbidities).

This study retains the inherent limitations of a retrospective design but attempted to overcome them as best as possible with thorough multivariable modeling using granular details (eg, type of chemotherapy, success of TURBT, and creatinine clearance) typically not found in large database studies. Owing to the retrospective nature of our analysis in conjunction with the variable documentation across VA hospitals and contracting with non-VA hospitals for radiation and/or chemotherapy administration, we were not able to report treatment interruptions or data on toxic effects. Both should be investigated further, ideally in a prospective fashion, because it would be helpful to gauge the effect of treatment interruptions on recurrence and survival outcomes while better educating providers and patients on the risks of CRT. Similarly, we were only able to classify the type of radiosensitizing chemotherapy and not able to quantify the number of cycles received. Although all of our patients received at least 1 cycle of chemotherapy, which we were able to subsequently classify as preferred or nonpreferred, the effect of the number of chemotherapy cycles during CRT is an important question for further research. Finally, although we do recommend the increased use of CRT, we recognize that social and economic issues more prominent in the US's hybrid-payer health care system compared with the VA's equal-access system likely have an additional role in the underuse of definitive treatment in MIBC and should be investigated further.^6^

In summary, this study's findings suggest that the vast majority of patients with MIBC treated with definitive-intent CRT are able to complete a definitive radiation regimen. With a low rate of treatment discontinuation, we posit that CRT is a feasible and promising definitive treatment option for patients with MIBC, especially the elderly and those with comorbidities. In addition to our identified predictors of discontinuation (poor renal function, incomplete TURBT, etc), further research is required to develop evidence-based guidelines for optimal patient selection. This study's findings, along with those of similar studies, should be taken into account as patients and clinicians navigate the challenges of treatment decision making for this aggressive disease.
